# Transcatheter aortic valve replacement 23 years after heart transplant for aortic insufficiency

**DOI:** 10.1186/s13019-023-02407-x

**Published:** 2023-10-07

**Authors:** Andrew Jones, Hossein Amirjamshidi, George Olverson, Frederick S. Ling, Kazuhiro Hisamoto

**Affiliations:** 1 University of Rochester School of Medicine and Dentistry, Rochester, NY USA; 2 Division of Cardiac Surgery, Department of Surgery, University of Rochester Medical Center, 601 Elmwood Ave, Box: Surg, Rochester, NY 14620 USA; 3 Department of Medicine, Cardiology, University of Rochester Medical Center, Rochester, NY USA

**Keywords:** Transcatheter aortic valve replacement, Heart transplantation, Aortic valve insufficiency, Aortic valve, Kidney transplantation

## Abstract

**Background:**

Clinicians continue to expand the availability of transcatheter aortic valve replacement (TAVR) for patients who historically would have been ineligible for surgical aortic valve replacement. Historically, reoperative aortic valve surgery after transplant was immensely complicated and high risk due to the repeat sternotomy approach, and the immunosuppression in transplant patients. As heart transplant patients continue to live longer, patients are beginning to develop novo aortic pathology of the transplanted organ. In these patients, TAVR may be a valuable rescue therapy for those with de-novo aortic valve disease.

**Case presentation:**

Here, we present a single case of a 70-year-old man with a history of heart transplant 23 years prior complicated by severe sternal infection and subsequent removal of his sternum. Additionally, this patient had a recent history of kidney transplant due to renal cell carcinoma necessitating nephrectomy. He subsequently developed progressive symptomatic aortic insufficiency and underwent a successful TAVR to treat his new aortic disease.

**Conclusions:**

To our knowledge, this represents only the second case report of TAVR for severe aortic insufficiency and one of the first reports of TAVR in a multiple organ recipient. TAVR may represent an important rescue therapy for post-transplant valve pathologies instead of high-risk reoperative surgical aortic valve replacement.

## Background

TAVR has emerged as a treatment option for patients with aortic valve disease. As TAVR techniques and tools have advanced, the indications for TAVR have broadened, offering symptom relief, and treatment options for patients who historically would not have been a candidate for surgical intervention. Furthermore, organ recipients have begun receiving TAVR with good results. TAVR has been described in heart transplant patients as early as 2010 [1], and has been attempted at increasing intervals from the original transplant, with cases showing success at 9 [1], 14 [2], and greater than 20 years after transplant in patients with aortic stenosis [3, 4]. Similarly, TAVR has begun to be used off-label to treat aortic insufficiency in similar patient populations with good results [5]. While ongoing questions remain about the comparative long-term efficacy of TAVR for aortic insufficiency when compared to surgical valve replacement [6], for select patients with significant surgical risk, early data show TAVR to be a viable option for patients with unacceptable surgical risk [7]. TAVR has also begun to be used in patients with multi-organ failure: Margale and Natani [8] presented a case of a successful TAVR after heart transplant in a patient with both HFrEF (heart failure with reduced ejection fraction), as well as stage 3 CKD (chronic kidney disease), but not yet in end-stage renal disease. TAVR has also demonstrated efficacy in patients with end-stage renal disease [8] and even after renal transplant [9]. Several studies have shown the efficacy and safety of TAVR in patients with kidney allografts [10]. These cases collectively demonstrate the efficacy of TAVR for complex patients with multi-organ pathology, which leads us to this case study. Here we present a single case of a 70-year-old man who had distantly undergone a heart transplant, and more recently undergone renal transplant who underwent TAVR with a balloon-expandable 29 mm aortic valve.

## Case presentation

**Fig. 1:**
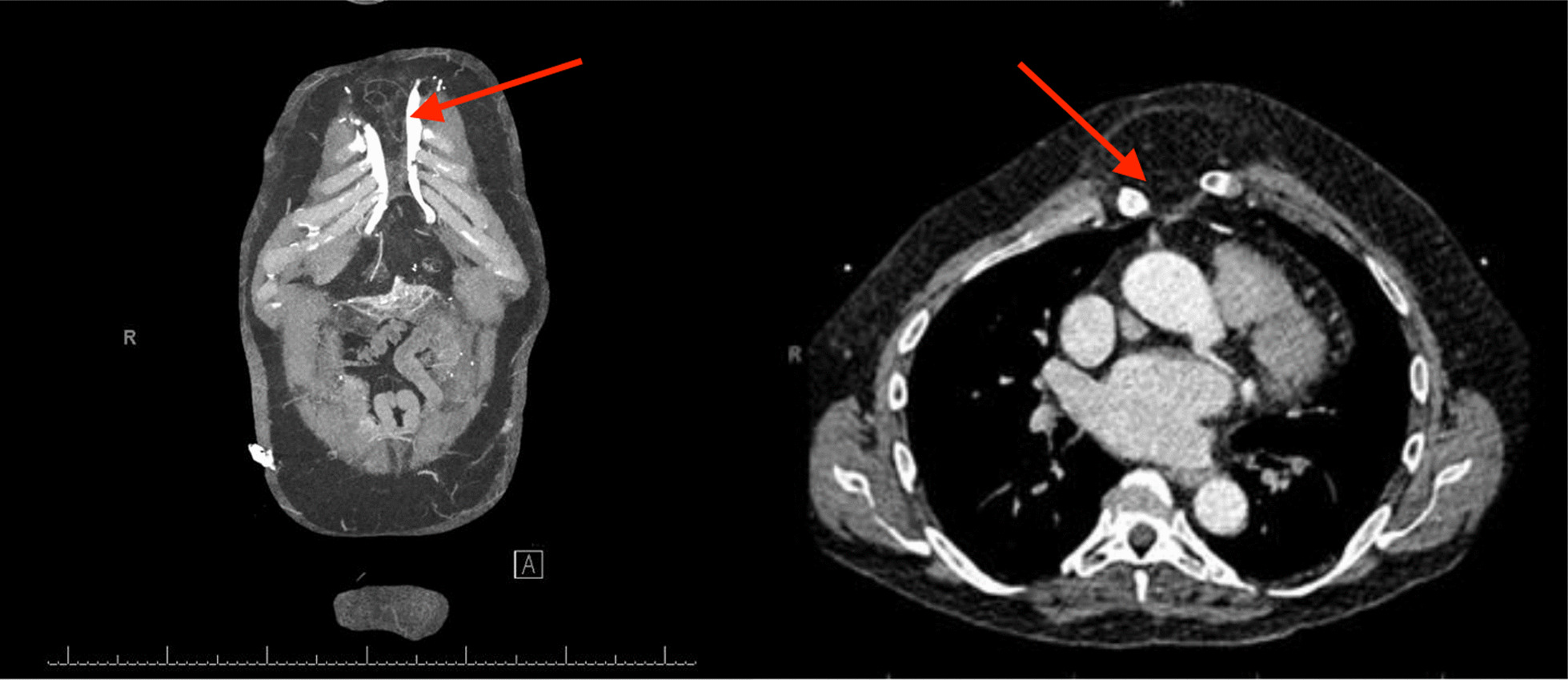
Pre-operative CT scan showing absent sternum

**Fig. 2:**
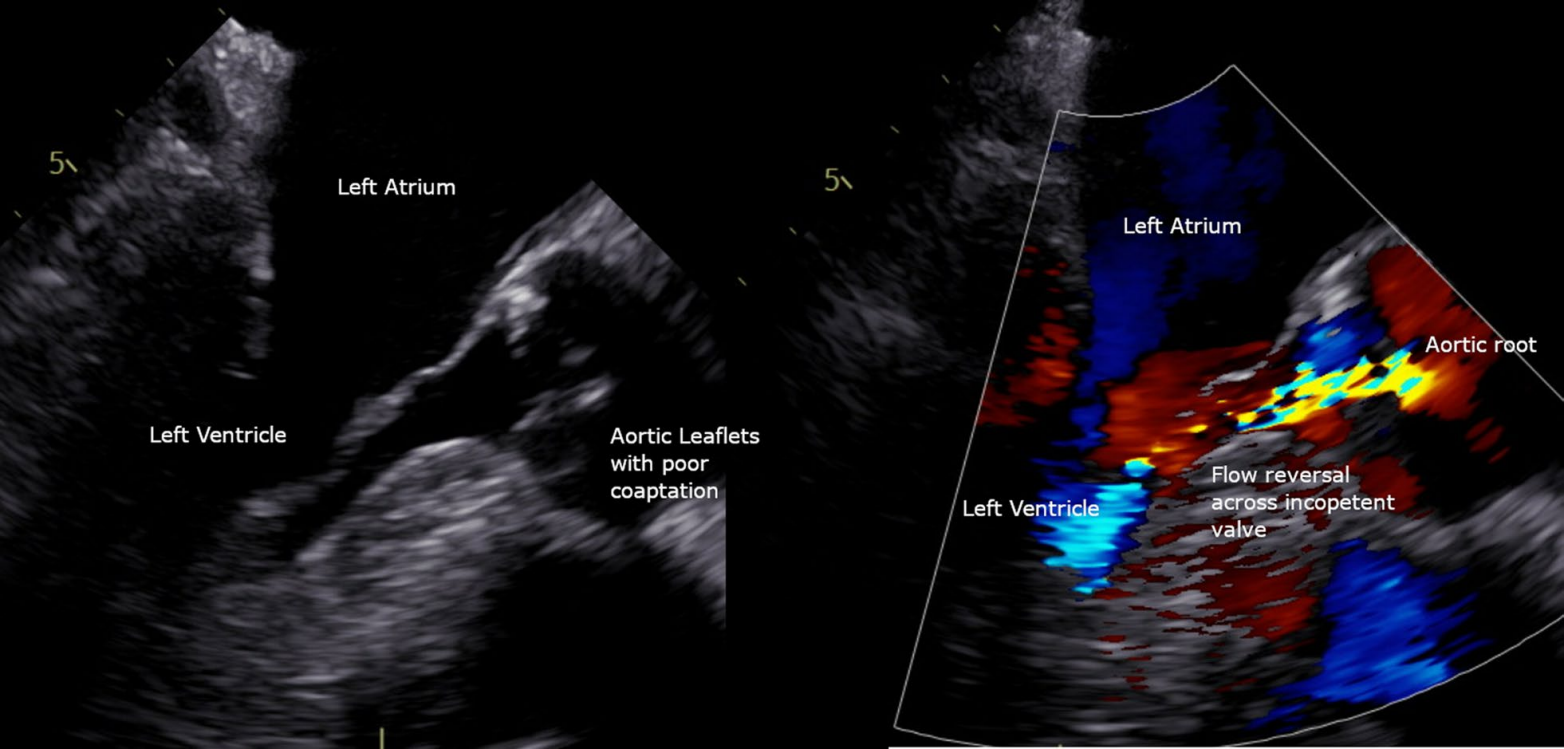
Preoperative Transesophageal Echocardiogram demonstrating insufficient aortic valve leaflets, with incomplete coaptation during diastole (left) and color doppler demonstrating flow reversal across the valve during diastole (right)

**Fig. 3:**
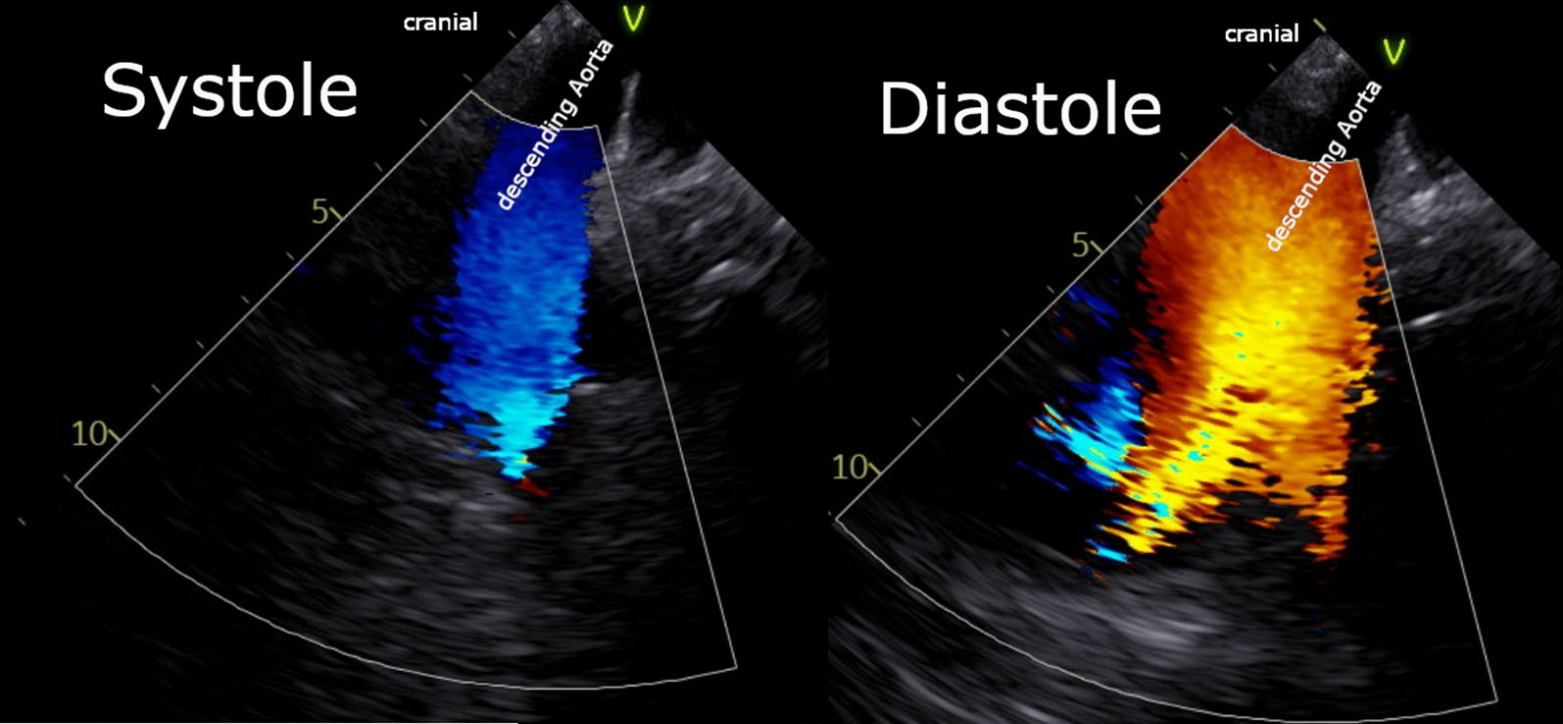
Preoperative Transesophageal Echocardiogram demonstrating significant flow reversal in the descending aorta during diastole (Right) as contrasted with the smooth systolic flow (Left)

**Fig. 4:**
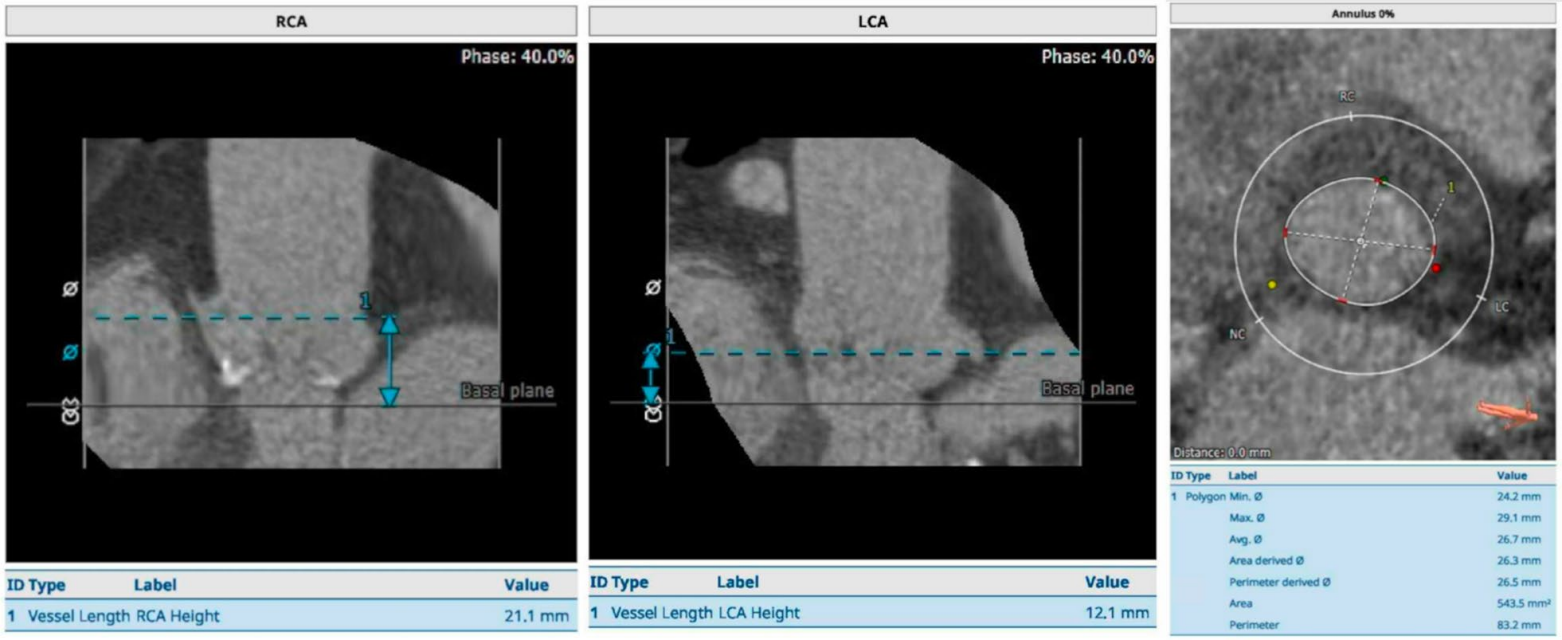
Pre-Operative CT scan showing anatomy of the transplanted aortic valve and root. The left and middle images demonstrate the left and right coronary heights, while the right image demonstrates the dimensions of the annulus

**Fig. 5:**
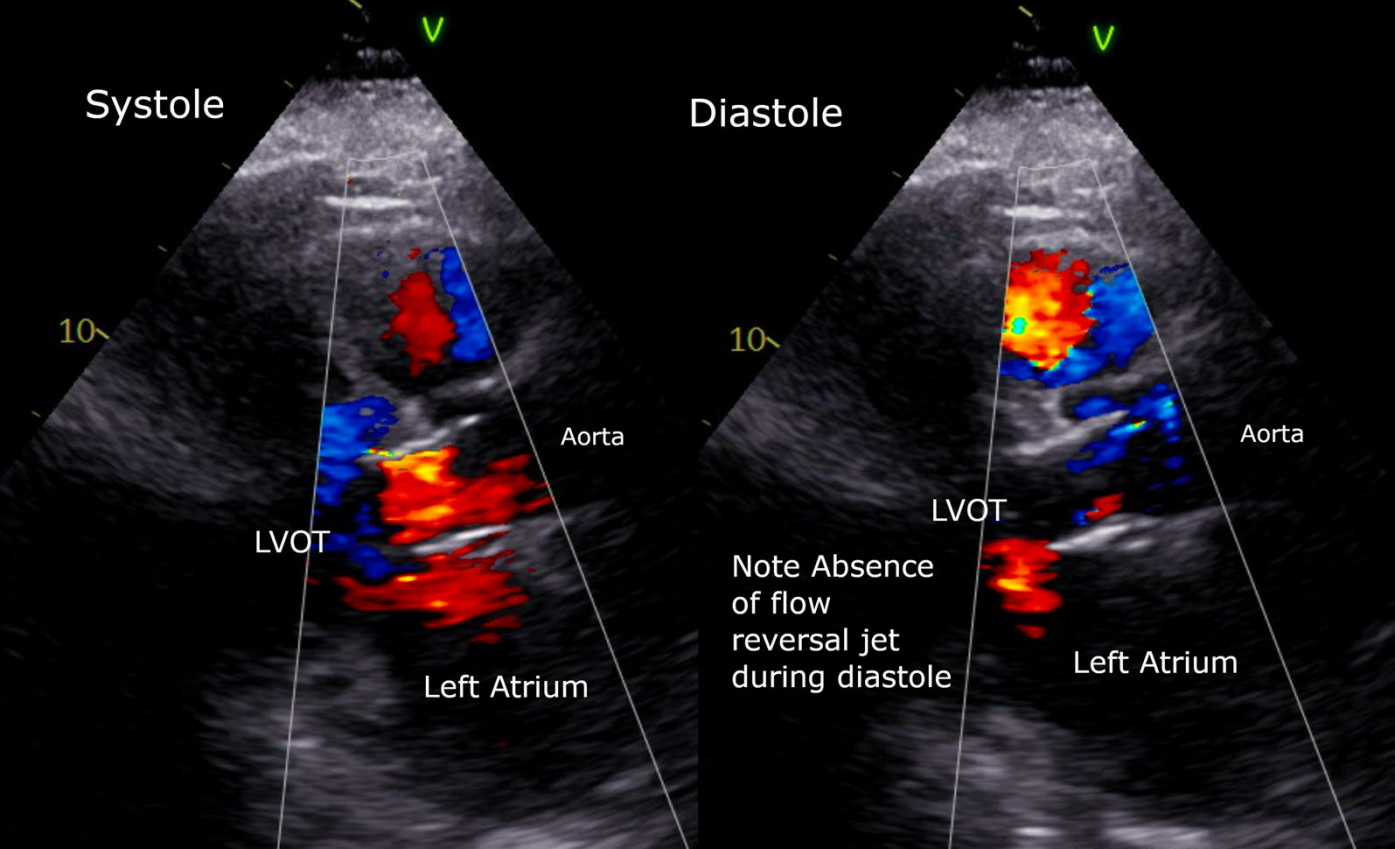
Post TAVR Transthoracic Echocardiogram showing good valve placement, and no residual paravalvular leak. Shown on the Left, Systolic flow through the valve and LVOT, and Right, no regurgitant jet, or paravalvular flow

This patient originally received his heart transplant after ischemic cardiomyopathy in 1999, which was complicated by deep sternal infection, resulting in sternal removal (Fig. 1). Later in 2013, he developed prostate cancer and underwent a prostatectomy. In 2018 he had end-stage renal disease and subsequently began peritoneal dialysis. In 2020, he was diagnosed with clear cell papillary renal carcinoma after undergoing nephrectomy for a renal mass. In 2021, he received a deceased donor allograft kidney. Aortic valve disease was originally identified in 2018 on a routine follow-up with his transplant center. He was noted to have aortic regurgitation at that time, which subsequently progressed to severe sclerosis and insufficiency with worsening regurgitation (Fig. 2) and diastolic flow reversal throughout the descending aorta (Fig. 3). As his aortic disease progressed, he developed exertional dyspnea at increasingly low levels of exertion. At that time, he was determined not to be a candidate for surgical aortic valve intervention due to his history of surgical infections with absent sternum, and high risk of complication. He received a preoperative CT scan of the heart which showed only mild calcification of a tricuspid aortic valve with an annular area of 543.5mm^2^ and an area-derived effective diameter of 26.3 mm, but circumference-derived diameter of 26.5 mm (Fig. 4 Right). The membranous septum was noted to be 9.8 mm. This scan also revealed only mild coronary artery disease in the left anterior descending, with a left coronary height of 12.1 mm (Fig. 4 Center), and a right coronary height of 21.1 mm (Fig. 4 Left). He was deemed suitable for TAVR via a transfemoral approach. We used coplanar fluoroscopy (LAO view) based on the preoperative CT and conventional deployment technique avoiding high deployment due to the height of the left coronary ostium and lack of annular calcification to reduce the risk of valve embolization. A 29 mm THV (transcatheter heart valve) was deployed under tachycardia pacing to allow for 20% oversizing to reduce the risk of valve embolization and PVL. The placement was confirmed with TEE and angiography which showed no paravalvular leak and transvalvular peak gradients of 11.7 mmHg, and a mean gradient of 7.7 mmHg without evidence of aortic regurgitation. Of note, after TAVR placement, the patient developed a new persistent left bundle branch block, with a 149 ms delay between ventricles, with no hemodynamic compromise. The patient was discharged the next day on mobile cardiac outpatient telemetry without any complications. At one month follow-up, his symptoms were significantly improved, and was able to walk over a mile each day. The left bundle branch block persisted but remained stable. On echocardiogram, the patient was found to have a peak aortic valve gradient of 16.4 mmHg, and a mean gradient of 9.4 mmHg without evidence of aortic regurgitation, or paravalvular leak. (Fig. 5) Two-month follow-up showed a peak gradient of 11.9 mmHg, and a mean gradient of 7.5 mmHg, with no paravalvular leak, and good valve position, with ongoing persistent left bundle branch block, but no other rhythm or hemodynamic changes. This study was granted an exception from full IRB approval, and consent was obtained from the patient for the publication of this information.

## Discussion and conclusions

We believe this to be the second case report of TAVR after transplant for severe aortic insufficiency. While other cases of TAVR after transplant for aortic insufficiency have been reported, we believe this to be the longest duration of TAVR for pure aortic insufficiency after a heart transplant. Additionally, while TAVR has been demonstrated in case studies in heart transplant patients, and renal transplant patients separately, this case demonstrates the potential for TAVR in complex patients with multi-organ transplants. As the management of heart transplant patients improves, and life expectancy expands, it is reasonable to consider TAVR for new and emerging aortic valve pathology in transplant recipients, even transplant recipients who have complex multi-organ transplants, or advanced renal disease.

We decided to use the lower profile BEV (balloon-expandable valve) instead of SEV (self-expanding valve) in part due to favorable operator experience with balloon-expandable valve in case of pure aortic insufficiency, and easier access to the coronary ostium if needed following valve deployment.

While TAVR is still not first-line therapy for aortic valve insufficiency, this patient’s comorbidities present an unacceptably high surgical risk. This case supports that TAVR may be a rescue option for patients who are not candidates for surgical valve replacement. While the new LBBB is a known side effect of TAVR, this patient did not exhibit many of the known risk factors for LBBB [11], and subsequently has not had any adverse events associated with this conduction abnormality. In the setting of these complicating disease processes, and the patient's immunosuppressed status the durability of the valve may be decreased, but as shown in this case report, TAVR may be considered in patients with multi-organ transplant as a viable intervention for aortic valve insufficiency if patient is not a candidate for surgical valve intervention.

## Data Availability

All data generated or analyzed during this study are included in this published article [and its supplementary information files].
